# Antibacterial Mechanism of Gloverin2 from Silkworm, *Bombyx mori*

**DOI:** 10.3390/ijms19082275

**Published:** 2018-08-03

**Authors:** Qian Wang, Pengchao Guo, Zhan Wang, Huawei Liu, Yunshi Zhang, Shan Jiang, Wenzhe Han, Qingyou Xia, Ping Zhao

**Affiliations:** 1 State Key Laboratory of Silkworm Genome Biology, Southwest University, Chongqing 400715, China; 15723012026@163.com (Q.W.); guopc@swu.edu.cn (P.G.); a918700210@email.swu.edu.cn (Z.W.); lhw888718@163.com (H.L.); swu_zhangys@163.com (Y.Z.); wanshan.jiang@foxmail.com (S.J.); 15903305805@163.com (W.H.); xiaqy@swu.edu.cn (Q.X.); 2 Chongqing Engineering and Technology Research Center for Novel Silk Materials, Chongqing 400715, China

**Keywords:** silkworm, antibacterial peptide, Gloverin, gram-negative bacteria, antibacterial mechanism

## Abstract

Gloverin is one of the glycine-rich antimicrobial peptide exclusively found in Lepidoptera insects. It is generally activated through the innate immune system in insects. In this study, recombinant Gloverin2 from *Bombyx mori* (BmGlv2) was synthesized using a prokaryotic expression system. Circular dichroism spectroscopy showed that the recombinant BmGlv2 has random coil structure, which is relatively stable at the temperatures ranging from 15 to 82.5 °C. Antimicrobial activity analysis revealed that BmGlv2 significantly inhibited the growth of gram-negative bacteria, *Escherichia coli JM109* and *Pseudomonas putida*, by disrupting cell integrity. Western blotting and immunofluorescence analyses suggested that BmGlv2 absorbed on the cell surface after incubation, which might be the first step in the antibacterial process. Our results also proved that the cell wall component lipopolysaccharides (LPS) induce a conformational change in BmGlv2 from a random coil to α-helix. Subsequently, α-helical BmGlv2 would recruit more BmGlv2 and form higher aggregation state. Collectively, these findings expand our understanding of antibacterial mechanism of BmGlv2.

## 1. Introduction

Silkworm (*Bombyx mori*) is an important economic insect and a classic model for studying Lepidoptera insects as well. Like other members of Lepidoptera, silkworms have an innate immune system and lack the acquired immune system [1,2]. The innate immune system of insects promptly responds to the infection by pathogenic microorganisms, activating immune pathways including Imd (Immune Deficiency) and Toll, and resulting in the production of antimicrobial peptides [3,4,5,6,7,8]. Gloverins (Glv) are glycine-rich antimicrobial peptides and effectors of innate immune responses exclusively present in Lepidopteran insects. Glv was first identified in *Hyposphora cecropia* (HcGlv) [9] and inhibits the growth of gram-negative bacteria *E. coli D21f2*. The inhibitory activity is not affected by heating at 100 °C for 10 min [9]. In hydrophilic environments, HcGlv mainly exists in a random coil structure and transitions into an α-helix-rich conformation in hydrophobic environment [9,10]. Previous studies have shown that Glv mainly exerts antibacterial effects on gram-negative bacteria, fungi and viruses [11,12,13]. Glv of *Helicoverpa armigera* showed activity against several *E. coli* that possess smooth lipopolysaccharides (LPS) [14]. Glv from *Plutella xylostella* showed high activity against *E. coli K12* [15] and *Trichoplusia ni* Glv is active against *E. coli D21f2*, *E. coli D22* strains and viruses. Only a few Glvs are known to exhibit inhibitory activity against gram-positive bacteria. For example, SeGlv in *Spodoptera exigua* can inhibit the growth of gram-positive bacterium (*Flavobacterium* sp.) [16] and MsGlv in *Manduca sexta* is active against gram-positive *Bacillus cereus* and fungi (*Saccharomyces cerevisiae* and *Cryptococcus neoformans*) but inactive against *E. coli* strains with smooth LPS [17].

Four Glv members have been identified in the silkworm, BmGloverin1, BmGloverin2 (BmGlv2), BmGloverin3 and BmGloverin4, which are famous marker genes in the immune response of the silkworm [18]. It was shown that the transcriptional level of BmGlvs can be strongly induced in the larval fat body by injecting of *E. coli JM109* [19]; BmGlv2 gene was up-regulated when the silkworm larvae were infected by *B. bassiana* [20]. Since different microorganisms induce different immune signaling pathways, BmGlvs are generally considered to be induced through the Toll or Imd pathways during the immune responses [21]. Similar to the HcGlv, BmGlvs also undergo a secondary structure transition from random coils to α-helices when incubated with commercial LPS [10]. Furthermore, BmGlv2 has a synergistic antifungal activity with *B. mori* cecropin A to against *B. bassiana* and the combined antimicrobial effect was stronger than the two alone [20], suggesting that these two antibacterial peptides might employ different antimicrobial strategies and cooperate to against the *B. bassiana.* Although numerous studies on the antimicrobial activity of Glvs have been reported and the pathways that induce Glvs are relatively well understood [21,22,23,24,25,26], the mechanism of action of BmGlv2 against microorganisms is still unclear. In this study, we expressed recombinant BmGlv2 and investigated its antibacterial properties. Structural and biochemical analyses suggested that BmGlv2 interacts with cell wall component LPS and accumulates on the surface of the bacterial cell through a conformational change. These findings led us to propose a putative model of BmGlvs against *E. coli* and provide clues for better understanding of the mechanism of action of Glvs.

## 2. Results

### 2.1. Sequence Analysis of BmGlv2

**Figure 1:**
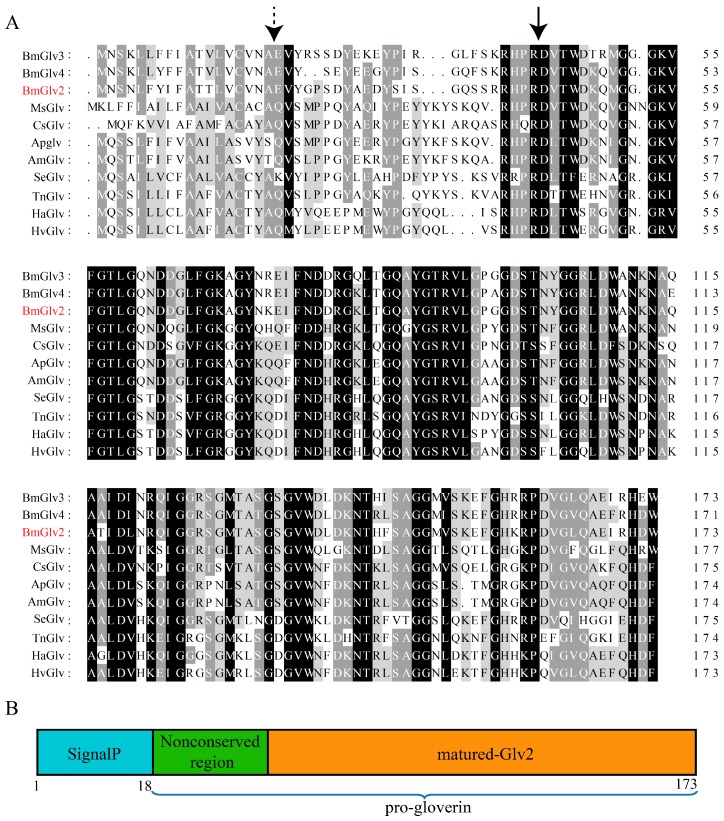
Sequence analysis of Glv in Lepidoptera. (**A**) Multiple sequence alignments of *Bombyx mori* BmGlv3 (NP_001093312.1), BmGlv4 (NP_001037684.1), BmGlv2 (NP_001037683.1), MsGlv from *Manduca sexta* (CAL25129.1); CsGlv from *Chilo suppressalis* (AHC94952.1); ApGlv from *Antheraea pernyi* (ACB45565.1); AmGlv from *Antheraea mylitta* (ABG72699.1); SeGlv from *Spodoptera exigua* (AKJ54490.1); TnGlv from *Trichoplusia ni* (AAG44367.1); HaGlv from *Helicoverpa armigera* (ALT16898.1); HvGlv from *Heliothis virescens* (ACR78446.1). The signal peptide cleavage side was marked with dotted arrow; and the mature Glv active site was marked with the solid arrow. Alignments were performed with Clustalx. Conserved, highly conserved and identical amino acid residues are highlighted in light gray, gray and black, respectively. (**B**) A diagram to show the organization of BmGlv2 Signalp, signal peptide.

The BmGlv2 cDNA encodes a 173 amino acid protein with a signal peptide composed of 18 amino acid residues, indicating that BmGlv2 might be secreted into the extracellular milieu. The molecular weight of full-length BmGlv2 is 16.8 kDa and the isoelectric point is 6.5. Protein blast analysis showed that BmGlv2 shares 74–86% identity with other BmGlvs in silkworm and a 52–62% identity with Glv from other Lepidoptera insects (Figure 1A). Multiple sequence alignment showed that Glvs consist of a signal peptide sequence, an N-terminal non-conserved region and a C-terminal conserved domain (Figure 1B). The matured BmGlv2 was predicted to be 14.1 kDa with the theoretical isoelectric point of 7.03. Moreover, the C-terminal region of Glvs is glycine-rich and the most conserved region, indicating that the active site of Glv may be located in this region.

### 2.2. Prokaryotic Expression and Thermal Stability Analysis of BmGlv2

**Figure 2:**
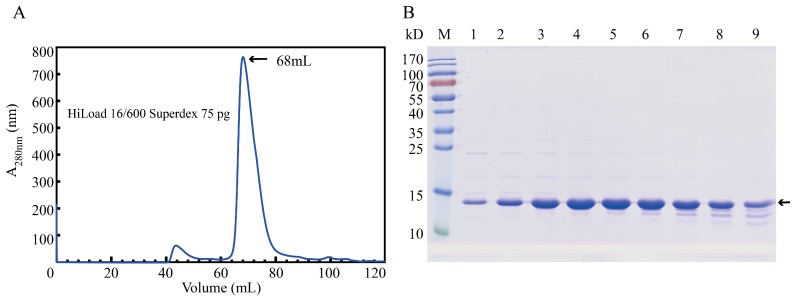
Purification of the recombinant BmGlv2. (**A**) Purification of the recombinant BmGlv2 by gel filtration. (**B**) SDS-PAGE analysis of the purified BmGlv2 protein. M: marker, 1 to 9: the fractions of elution peak.

To obtain BmGlv2 for inhibitory and biochemical analyses, we expressed soluble BmGlv2 in *E. coli* BL21 (DE3) under the induction of 0.1 mM isopropyl β-D-1-thiogalactopyranoside (IPTG) at 37 °C for 4 h and purified the protein by metal chelated affinity chromatography and gel filtration chromatography. The purity was confirmed by sodium dodecyl sulfate–polyacrylamide gel electrophoresis (SDS-PAGE). Gel filtration analysis showed that the eluted peak of recombinant BmGlv2 is corresponding to ~42.3 kDa (Figure 2B), which is approximately equal to three times its theoretical molecular weight of 14.1 kDa. This finding indicated that BmGlv2 exists as a trimer in solution.

**Figure 3:**
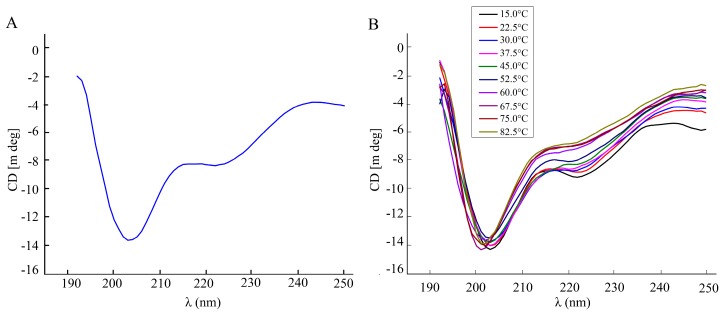
The secondary structure and thermal stability analysis of BmGlv2. (**A**) Circular dichroism spectrum of BmGlv2. (**B**) The secondary conformational profiles of BmGlv2 in the range of 15 to 82.5 °C.

The secondary structure of recombinant BmGlv2 was analyzed by far-UV circular dichroism spectroscopy (CD). As shown in Figure 3A, a significantly negative band at 200 nm and a weakly negative band at 222 nm appeared, indicating BmGlv2 had a primarily random coil structure. To explore the effects of temperatures on BmGlv2 structure, the thermal stability of protein was monitored by a shift in CD spectra. As shown in Figure 3B, with the increase of temperature, the weak band corresponding to α-helix reduced gradually, while the mainly negative band was near 200 nm, indicating that BmGlv2 structure was relatively stable in the range of 15 to 82.5 °C (Figure 3B).

### 2.3. Antibacterial Activity of BmGlv2

**Figure 4:**
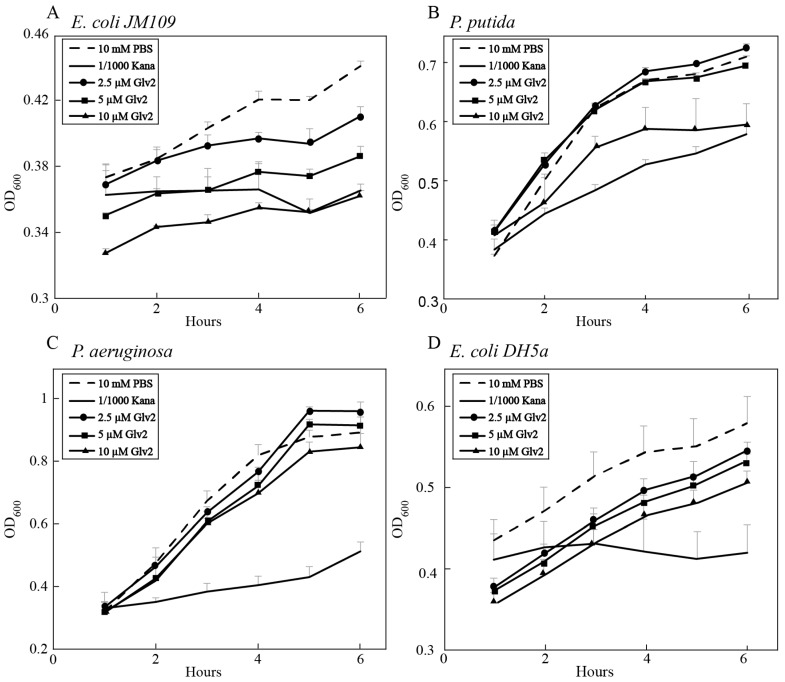
Antibacterial activity of BmGlv2. (**A**) Inhibitory effect of BmGlv2 on the growth of *E. coli JM109*. (**B**) Inhibitory effect of BmGlv2 on the growth of *P. putida.* (**C**) Inhibitory effect of BmGlv2 on the growth of *P. aeruginosa.* (**D**) Inhibitory effect of BmGlv2 on the growth of *E. coli DH5a*.

To evaluate the inhibitory activity of BmGlv2 on gram-negative bacteria, the *E. coli JM109*, *P. putida*, *P. aeruginosa* and *E. coli DH5a* were incubated with different concentrations of recombinant BmGlv2. The growth curve of bacteria was monitored by ultraviolet spectrophotometry. As shown in Figure 4A,B, BmGlv2 was active against the *E. coli JM109* and *P. putida* at the concentration of 10 µM concentration. Significant inhibitory activity against *E. coli JM109* was observed even at low concentrations (2.5 µM) of BmGlv2 (Figure 4A). BmGlv2 did not have any significant antibacterial activity against *P. putida* at low concentrations (2.5 and 5 µM), suggesting BmGlv2 has a higher binding affinity to *E. coli JM109* than to *P. putida*. Moreover, BmGlv2 was inactive against *P. aeruginosa* (Figure 4C) and *E. coli DH5a* (Figure 4D).

**Figure 5:**
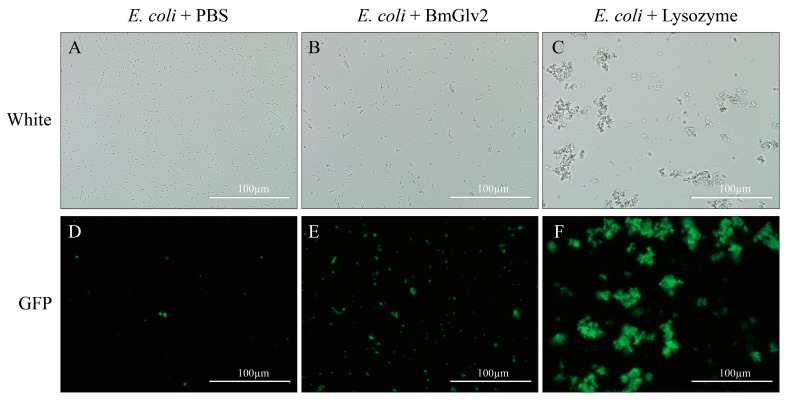
The lethal activity of BmGlv2. (**A**–**C**) The images under bright light. (**D**–**F**) The images under fluorescent light. The scale bar was marked in the picture. PBS was used as a negative control and the lysozyme was used as a positive control.

We also confirmed the antibacterial activity of BmGlv2 against *E. coli JM109* using bacterial viability assay (Figure 5). Under bright light, *E. coli* cells incubated in phosphate buffer saline (PBS) were adequately distributed and maintained cellular integrity (Figure 5A), while the *E. coli* cells incubated with BmGlv2 aggregated into clusters and were partially broken (Figure 5B). Moreover, almost 40% of cells in the BmGlv2-treatment group stained green with Sytox staining while no cells in the PBS group took up the stain. Ninety percent of cells in the lysozyme-treatment group were stained (Figure 5E,F), indicating that these cells were dead. These finding suggested that BmGlv2 causes cell breakage and cell death in *E. coli JM109*.

### 2.4. Binding Efficiency of BmGlv2 to E. coli JM109

**Figure 6:**
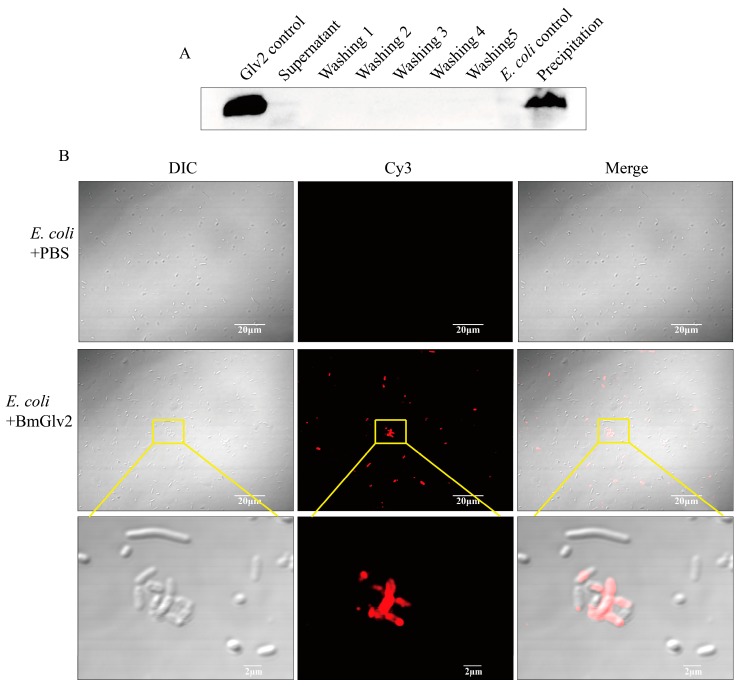
The binding activity analysis of recombinant BmGlv2. (**A**) Western Blotting analysis of the binding of BmGlv2 to *E. coli JM109* cells. Glv2 control, recombinant Glv2; Supernatant, a supernatant fraction after binding incubation; Washing1–Washing5, the first to fifth washing collection; *E. coli* control, *E. coli JM109* only; Precipitation, the centrifuged *E. coli* cells after incubation and washing. The *E. coli JM109* was incubated with BmGlv2 for 3 h. (**B**) Immunofluorescence localization analysis of BmGlv2 on the surface of *E. coli JM109* cells. Confocal microscopy was performed to visualize BmGlv2 binding (red) on the cell walls of *E. coli JM109*. The third row amplified from the second row described the detail of BmGlv2 on the surface of *E. coli JM109* cells. The PBS was used as a negative control. The scale bar was marked in the picture.

Since recombinant BmGlv2 showed a significant inhibitory effect against *E. coli JM109*, we determined the binding efficiency of BmGlv2 with *E. coli JM109*. After incubation with BmGlv2 and repeated washing with PBS, the BmGlv2 signal was observed in the cell fraction, while no signal was detected in the supernatant and washing fractions by Western blotting (Figure 6A), indicating that BmGlv2 proteins were fully bound to live *E. coli* cells. Furthermore, immunofluorescence analysis showed that BmGlv2 signals were observed on *E. coli JM109* (Figure 6B), suggesting the BmGlv2 was located on the *E. coli JM109* cell surface. Interestingly, BmGlv2 signals tended to accumulate on the cell surface, which might relate to its antimicrobial mechanism. These binding analyses revealed that BmGlv2 could be adsorbed on *E. coli* cell surface during the incubation, which probably is the first step in the antibacterial process. 

### 2.5. Interaction between BmGlv2 and LPS

**Figure 7:**
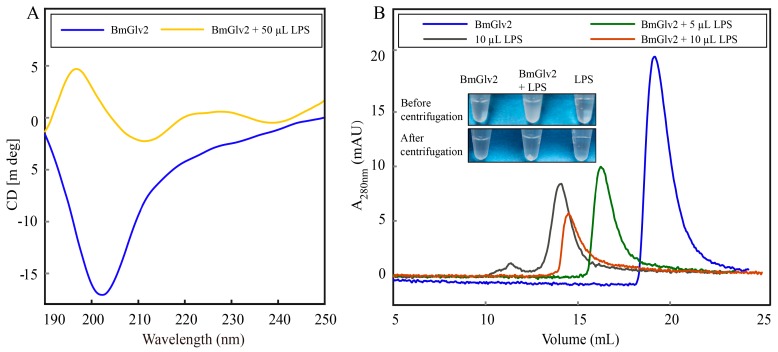
Interaction analysis of BmGlv2 and LPS from *E. coli JM109*. (**A**) CD analysis of BmGlv2 in the presence or absence of LPS. (**B**) Gel filtration analysis of BmGlv2 with the amounts increasing of LPS. HiLoad Superdex 75 10/300 GL was used and the LPS was extracted from *E. coli JM109*.

Previous studies have reported that Glv may interact with the lipid A moiety of LPS to exert antibacterial activity [9,17]. To explore the binding and synergistic relationship between BmGlv2 and natural LPS, we extracted LPS from *E. coli JM109* by LPS extraction kit and then incubated it with BmGlv2. CD was performed to analyze the structural transition of BmGlv2. As shown in Figure 7A, BmGlv2 possesses primarily random coil structure and underwent a transition to an α-helical conformation in the presence of LPS, indicating that hydrophobic substrate LPS promoted the conformational change of BmGlv2. To detect the interaction of BmGlv2 and LPS, different concentrations of LPS were added to BmGlv2. After incubation for 30 min, precipitation was observed, which increased with the increasing concentrations of LPS. SDS-PAGE and Western blotting results showed that both BmGlv2 and LPS were present in the precipitate (Figure 7B). The supernatant of the mixture was analyzed by gel filtration chromatography (HiLoad 10/300 Superdex 75 column). As shown in Figure 7B, BmGlv2 only was eluted at about 18 mL and the LPS only was eluted at about 12 mL but no BmGlv2-LPS complex peak (faster than 12 mL) was detected. Notably, only one elution peak was observed in the supernatant of BmGlv2 and LPS mixture, which contained BmGlv2 only but eluted faster than BmGlv2. Moreover, the mixture peak progressively reduced and shifted from right to left with increasing concentration of LPS, implying BmGlv2 progressively formed higher aggregation state in the environment of LPS and that aggregated BmGlv2 could interact with LPS and form precipitate with higher concentrations of LPS. These findings suggested that the cell wall component LPS promotes the conformational change in BmGlv2 from random coil to α-helix and the α-helical BmGlv2 favorably binds to LPS, Subsequently, the α-helical BmGlv2 would recruit more BmGlv2 and form higher aggregates.

## 3. Discussion

Similar to other Glv homologs, the positively charged BmGlv2 consists of a signal peptide sequence, N-terminal non-conserved region and C-terminal conserved domain. The C-terminal conserved domain represents the matured Glv (active form), which is normally activated by other innate immune factors in vivo [3]. To obtain active BmGlv2 and investigate its antibacterial properties, we expressed the mature BmGlv2 in *E. coli*. Our studies found that the activity of recombinant BmGlv2 was different towards different gram-negative bacteria. Gram-negative bacteria have different types of LPS, smooth or rough. The presence or absence of the *O*-polysaccharide chain (the outermost domain of the LPS) determines whether the LPS is rough or smooth. A long *O*-polysaccharide chain would render the LPS smooth, whereas the absence or reduction of *O*-antigen chain would make LPS rough [27]. Previous studies proved that BmGlvs could inhibit the growth of gram-negative bacteria with rough LPS but cannot inhibit the growth of gram-negative bacteria with smooth LPS [10]. The LPS in the *E. coli JM109*, *E. coli* DH5α, *P. putida* and *P. aeruginosa* probably contains different *O*-polysaccharide chains and this might underlie the different sensitivities to BmGlv2.

The antibacterial effect towards *E. coli JM109* was further confirmed by Sytox green staining. Sytox green is a nucleic acid stain for distinguishing dead cells from live cells. It was found that almost half of *E. coli* cells were stained in the BmGlv2-treated group compared to that of the PBS-treated group, indicating these *E. coli* cells were killed by BmGlv2 (Figure 5B,E). Considering that BmGlv2 is a kind of biomacromolecule with a molecular weight of 14 kDa, which cannot shuttle into the cell and exerts its antibacterial activity in the cytoplasm directly, we deduced BmGlv2 acts exclusively on the *E. coli* surface and killed *E. coli* by disrupting cell integrity. *E. coli* cells incubated with BmGlv2 were partially broken and aggregated under the bright light (Figure 5 and Figure 6). Western blotting and immunofluorescence staining confirmed that BmGlv2 could bind on the surface of the bacterial cell after incubation with *E. coli JM109*. Similar observations of binding activity were found in the antibacterial assay of AcVSPI [28] and BmCPT1 [29], which suggested that both inhibitors were located on the bacterial cell walls. Thus, our findings suggest that antimicrobial peptides absorbing on the surface of the bacterial cell is a canonical process against bacteria, which might be the necessary step of antibacterial process. In addition, the outer membrane of the cell wall in gram-negative bacteria, which is exposed to outside and is composed of the outermost layer of LPS and lipid bilayer. That is why researchers generally select LPS and membrane-like components as a substrate to study the binding activity of Glvs. In the presence of natural LPS, BmGlv2 underwent a conformational change from random coil to a-helix (Figure 7A). A similar conformational change is reported in the Glv of *Hyalophora* [9]. In addition, hydrophobic substrates, such as SDS, Hexafluoroisopropanol and dodecylphosphocholine also caused the conformational transition from random coil to α-helix [9,10], suggesting that both LPS and lipid bilayer of the cell wall in gram-negative bacteria are involved in the conformational transition of Glv. Most antibacterial peptides, including defensins and bacteriocins except some β-sheet antibacterial peptides, exert their activity through their α-helices in a membrane-like environment [30,31]. The α-helix amphipathic secondary structure is important for insertion into the bacterial membrane [32]. In our study, gel filtration chromatography analysis showed that BmGlv2 progressively aggregated with increasing amounts of LPS (Figure 7B), indicating that the α-helical BmGlv2 interacted with LPS recruiting more BmGlv2 and forming higher aggregates in the membrane-like environment. Both our results and previous reports revealed that BmGlv2 and other BmGlvs could interact with rough LPS and not smooth [10], In addition, most Glvs could inhibit the growth of bacteria with rough LPS but not the bacteria with smooth LPS, indicating the rough LPS is vital for antibacterial activity. Moreover, Axen et al. have proved that adding LPS would decrease the inhibitory effect of recombinant Glv against growth of *E. coli* and the effect was dose-depended [9], further suggesting that the binding of Glv2 with LPS is necessary for its antibacterial activity. However, to confirm the importance of the binding of BmGlv2 with LPS, the antibacterial activity of mutant Glv2, which cannot bind to LPS, should be detected and explored in the further studies. 

Based on these findings and previous studies, we proposed a mechanism for the action of BmGlv. First, the positively charged BmGlv is attracted to the gram-negative bacteria by electrostatic attraction. Then, the LPS and lipid bilayer promotes conformational changes in the attached BmGlv inducing a transition from random coil to α-helix. The α-helical BmGlv2 interacts with the outer membrane component LPS, which might be an essential process that BmGlv exerts antibacterial activity. α-helical BmGlv2 then recruits more BmGlv2 to form aggregated BmGlv clusters on the cell wall. The amphipathic conformation permits BmGlv to insert into the hydrophobic sector of the lipid bilayer, disrupting the stability and increasing the permeability of the bacterial cell wall. Finally, the α-helical structure drives the BmGlv2 deeper into the cytoplasmic membrane, causing cell rupture and death in *E. coli*. Further studies are required for a clear understanding of how Glv2s disrupt the cell wall and create cell wall pores, inducing cell death. This mechanism may be exploited in generating novel species-specific antibacterial drugs.

## 4. Materials and Methods

### 4.1. Sequence Alignment

The Lepidopteran Glvs nucleotide sequences and amino acid sequences were downloaded from the silkworm genome database SilkDB (available online: http://silkworm.swu.edu.cn/silkdb) and NCBI (available online: https://www.ncbi.nlm.nih.gov/). The sequences of Glvs were aligned using the clustax and GenDoc. The TMHMM Server v.2.0 (available online: http://www.cbs.dtu.dk/services/TMHMM/) and SignalP 4.1 Server (available online: http://www.cbs.dtu.dk/services/SignalP/) predicted the transmembrane helices and signal peptide of BmGlv protein. Primer premier 5.0 was used to design primers for *BmGlv2*. The ExPASy website (available online: https://web.expasy.org/compute_pi/) was used to predict the protein molecular weight and isoelectric point of BmGlv2. 

### 4.2. Cloning of BmGlv2

The diapause silkworm strain (*Dazao*) was provided by the Gene Resource Library of Domesticated Silkworm (Southwest University, Chongqing, China). The primers containing *Xho I* and *Nco I* sites were designed based on the conserved coding region of the *BmGlv2* (CDS: BGIBMGA005658-TA) sequence of silkworm. The gene of *BmGlv2* with complete ORF was cloned by the fat body cDNA of silkworm at day 3 of the 5th instar using the primers. Then the PCR products were ligated into the pMD-19T Vector (Takara, Tokyo, Japan). The verified correct recombinant plasmid *BmGlv2*-pMD-19T-simple and pET-21d vector was digested with *Nco I* and *Xho I*, then the *BmGlv2*-pET21d recombinant expression plasmid was constructed by ligation. The recombinant plasmid was confirmed by restriction and DNA sequencing.

### 4.3. Expression and Purification of BmGlv2

The *BmGlv2*-pET21d was transformed into *E. coli* Bl21 (DE3) cell and the positive bacterial colonies were inoculated into LB medium containing ampicillin (100 μg/mL) at 37 °C to OD_600_ = 0.6 to 0.8 and then protein expression was induced by 0.1 mM IPTG at 37 °C for 5 h. The bacterial cells were harvested by centrifugation at 6000× *g* for 20 min at 4 °C and then re-suspended in lysis buffer (20 mM Tris-HCl, 200 mM NaCl, pH 8.0).

After 6 min of sonication and centrifugation at 12,000× *g* for 25 min, the supernatant was collected and applied to Ni^2+^-NTA column (GE Healthcare, Chicago, IL, USA) equilibrated with binding buffer (20 mM Tris-HCl, pH 8.0, 200 mM NaCl). The target protein was eluted with 350 mM imidazole in 20 mM Tris-HCl, 200 mM NaCl, pH 8.0 and then was loaded onto a HiLoad 16/60 Superdex 200 column (GE Healthcare) pre-equilibrated with 10 mM phosphate, pH 6.0. The purified protein was estimated by SDS-PAGE then stored at −80 °C.

### 4.4. Circular Dichroism Spectroscopy

CD spectra were recorded on a MOS-500 circular dichroism spectrometer (Bil-Logic, Seyssinet-Pariset, France) with a 0.1 cm quartz cell at 25 °C in 190–250 nm using standard procedures. BmGlv2 concentration was 0.05 mg/mL in 10 mM phosphate, pH 8.0. The effects of temperature on the secondary structure of BmGlv2 were performed from 15 to 82.5 °C at a step of 2.5 °C. The concentration was 0.05 mg/mL. The percentages of secondary structure elements were estimated with the method of variable selection using a data base of 22 proteins with known secondary structure [33,34].

### 4.5. Antibacterial Activity Assay

Antimicrobial activity of recombinant BmGlv2 was estimated against four gram-negative bacterial *E. coli JM109*, *P. putida*, *P. aeruginosa* and *E. coli DH5α*. After cultured in LB medium to OD_600_ of 0.4, the bacteria (160 μL) were incubated with 40 μL recombinant protein BmGlv2 (final concentration of 2.5, 5, 10 µM) in a 96-well plate at 37 °C with 80 rpm. The ultraviolet spectrophotometer (ultrospec 10, Amercham Biosciences, Tianjin, China) was used to measure the OD_600_ value every hour.

To test the lethal effect of BmGlv2 to *E. coli JM109*, fluorescent staining was performed. The recombinant BmGlv2 protein (10 µM) was incubated with *E. coli JM109* (OD_600_ = 0.4) at 25 °C for 3 h with the shaking of 80 rpm. Set the BmGlv2 only and the *E. coli* only as a control group. The samples were washed 3 times and re-suspended with 200 µL PBS, pH 6.0, fluorescent staining was added according to the Sytox Green [35] (Thermo Fisher Scientific, Waltham, MA, USA) instructions and then observed with a fluorescence microscope.

### 4.6. Microbial Binding Assay

Gram-negative bacterial *E. coli JM109* was used in this study. The ability of BmGlv2 binding with bacteria was determined by Western blotting and immunofluorescence staining. The bacteria were cultured in LB medium to OD_600_ of 0.4. After centrifuging at 6000× *g* for 3 min and washing 3 times with washing buffer (10 mM PBS, pH 6.0), bacteria were re-suspended with 200 µL binding buffer (10 mM PBS, pH 6.0). The recombinant BmGlv2 (final concentration of 10 μM) 40 μL and 160 μL were mixed in a 1.5 mL centrifuge tube and incubated at room temperature for 3 h. After centrifuging at 3000× *g* for 3 min, the bacterial pellets were washed 5 times to remove the uncombined recombinant protein. Then 15% SDS-PAGE gel electrophoresis and Western blotting (anti-BmGlv2 tag rabbit monoclonal antibody as the primary antibody) analyzed the combining effect. For immunofluorescence staining assay, the *E. coli JM109* cell treated by BmGlv2 was incubated with the normal goat serum (with final concentration of 5%, Solarbio, Beijing, China) at 4 °C overnight. After washing 3 times with 10 mM phosphate, pH 6.0, the microorganism samples were incubated with BmGlv2 rabbit antibody (diluted 1:500 (*v*/*v*) with 10 mM phosphate, pH 6.0) and then incubated with fluorescein-conjugated goat anti-rabbit antibody Cy3 (diluted 1:500 (*v*/*v*) with 10 mM phosphate, pH 6.0). The recombinant BmGlv2 peptides on the bacterial cell surfaces were visualized using laser-scanning confocal microscopy (Carl Zeiss LSM 510, Zeiss, Jena, Germany).

### 4.7. LPS Bingding Assay

The circular dichroism spectral and gel filtration chromatography was used to explore the interaction between BmGlv2 and LPS of *E. coli JM109*. The 50, 10, 5 μL *E. coli JM109* LPS (with initial concentration of 1 µg/µL theoretically) extracted by LPS extraction kit (iNtRon, Gyeonggi-do, Korea) was mixed with 150 μL recombinant protein BmGlv2 (with a final concentration of 10 μM) respectively. After incubation at 25 °C for 3 h, the secondary structure of BmGlv2 was performed by circular dichroism spectral. Spectra were recorded over a wavelength from 190 to 260 nm. Each spectrum was obtained by subtracting the signal from protein-free solution. Simultaneously, the incubated mixtures of BmGlv2 and LPS were loaded onto a HiLoad 10/300 Superdex 75 column (GE Healthcare) pre-equilibrated with 10 mM phosphate, pH 6.0 and the eluted sample was estimated by Western blotting.
